# Genome-Wide Identification of the *PP2A* Gene Family in Strawberry (*Fragaria* × *ananassa*) and Pharmacological Evidence for the Involvement of PP2A in ALA-Mediated Salt Tolerance

**DOI:** 10.3390/genes17101213

**Published:** 2026-09-30

**Authors:** Youyue He, Yuhua Wang, Renyong Cao, Xinghui Li

**Affiliations:** 1 College of Horticulture, Nanjing Agricultural University, Nanjing 210095, China; 2022204005@stu.njau.edu.cn (Y.H.); wangyuhua@njau.edu.cn (Y.W.); 2 College of Landscape Architecture, Jiangsu Vocational College of Agriculture and Forestry, Jurong 212400, China

**Keywords:** *Fragaria* × *ananassa*, protein phosphatase 2A, 5-aminolevulinic acid (ALA), salt stress, *PP2A* family, antioxidant enzyme

## Abstract

**Background**: Protein phosphatase 2A (PP2A) is a conserved serine/threonine phosphatase that plays crucial roles in stress responses, yet its function in strawberry (*Fragaria* spp.) remains largely uncharacterized. This study aimed to characterize the *PP2A* gene family in octoploid strawberry and to investigate its involvement in ALA-induced salt tolerance. **Methods**: We identified *PP2A* genes in the octoploid strawberry ‘Benihoppe’ genome and classified them into scaffold (*A*), regulatory (*B55*, *B56*, *B″*), and catalytic (*C*) subunit families. Segmental duplication and selection pressure were analyzed using Ka/Ks. Time-course RNA-seq was performed under NaCl and ALA treatments. Physiological assays measured SOD and CAT activities, O_2_•^−^ and MDA accumulation, and PP2A activity. Cantharidin was used to pharmacologically inhibit PP2A. **Results**: Seventy-five *PP2A* genes were identified. Segmental duplication was the primary driver of *PP2A* family expansion (70/75 genes), with all duplicated pairs under strong purifying selection (mean Ka/Ks = 0.138). Time-course RNA-seq analysed with DESeq2 identified three B56-subfamily *PP2A* genes as differentially expressed between NaCl and NaCl + ALA at 24 h (FDR < 0.05), the most strongly affected being *FaPP2AB′η-5*; in contrast, several C4 catalytic subunits were suppressed by salt stress. This ALA-responsive induction declined sharply at 48 h and 72 h. Physiological assays demonstrated that ALA significantly enhanced SOD and CAT activities while reducing O_2_•^−^ and MDA accumulation under NaCl stress, and ALA also elevated PP2A activity. Cantharidin largely attenuated both the ALA-induced increase in PP2A activity and ALA-mediated antioxidant protection. **Conclusions**: This study provides a comprehensive characterization of the strawberry *PP2A* gene family and indicates that PP2A activity contributes to ALA-induced salt tolerance, revealing a functional link between ALA signaling and PP2A-mediated antioxidant defense.

## 1. Introduction

Soil salinization affects over 800 million hectares of arable land worldwide, representing one of the most severe abiotic constraints on crop productivity [1]. Strawberry (*Fragaria* × *ananassa*) is an economically important fruit valued for its flavor and nutritional properties, with global production exceeding 9 million tons annually. It can be cultivated in open fields, greenhouses, and intercropping systems, making it a highly adaptable economic crop. However, strawberry is classified as salt-sensitive, with yield losses of 20–40% under moderate salinity [2,3]. As an octoploid crop (2n = 8x = 56) formed through sequential hybridization of four diploid progenitor species, the cultivated strawberry possesses a highly duplicated and subgenome-partitioned genome that provides a unique resource for gene family expansion and functional diversification [4]. The complexity of the octoploid genome, while challenging for genetic analysis, offers an exceptional opportunity to study how polyploidization drives the evolution of stress-responsive gene families.

Plants have evolved complex regulatory networks to cope with salt stress, among which reversible protein phosphorylation plays a central role in signal transduction and metabolic regulation [5]. The classic Salt Overly Sensitive (SOS) pathway exemplifies how phosphorylation cascades govern ion homeostasis under salinity [6], while mitogen-activated protein kinase (MAPK) cascades [7] and calcium-dependent protein kinases (CDPKs) [8] orchestrate transcriptional reprogramming. In contrast to the extensive characterization of protein kinases, the roles of protein phosphatases, which counterbalance kinase-mediated phosphorylation, have received considerably less attention. Among these, protein phosphatase 2A (PP2A) has emerged as a key regulatory hub in stress signaling, yet our understanding of its functions in *Rosaceae* crops remains fragmentary.

PP2A is a heterotrimeric holoenzyme composed of a scaffolding A subunit, a regulatory B subunit, and a catalytic C subunit [9]. In plants, the B subunit family is highly diversified and divided into B55, B56, and B″ (also known as PR72) subfamilies, among which B″ includes the plant-specific TON2/FASS proteins involved in cytoskeletal organization, with the number of B subunit genes far exceeding that of A and C subunits. This pattern contrasts with mammals and yeast and suggests that regulatory subunit expansion underlies the functional versatility of plant PP2A [10,11]. B regulatory subunits determine substrate specificity and subcellular targeting, and have been implicated in pathogen defense, flowering time control, and salt tolerance [11]. The C catalytic subunits, although fewer in number, are highly conserved across eukaryotes. In *Arabidopsis*, five catalytic subunits (*PP2A-C1* to *C5*) are divided into two clades: Clade I (*C1*, *C2*, *C5*) and Clade II (*C3*, *C4*) [12]. These two clades are functionally distinct, with Clade II members implicated in auxin transport and developmental processes. Recent genetic studies have demonstrated that the PP2A catalytic subunit *C5* positively regulates salt tolerance in *Arabidopsis* through interaction with vacuolar chloride channels, operating independently of the SOS pathway [13], while FERONIA kinase and PP2A form an antagonistic regulatory module that balances salt tolerance through mutual phosphorylation and dephosphorylation [14]. Genome-wide analyses of *PP2A* families have been reported in several plant species, including *Arabidopsis* (26 members), rice (30), tea [15], rubber tree [16], and soybean [17], revealing substantial lineage-specific expansion driven by whole-genome duplication events [18]. Within *Rosaceae*, the *PP2A* gene family has recently been characterized in apple, where 42 members were identified and ALA was shown to upregulate *MdPTPA* to activate *MdPP2AC* and promote stomatal opening [19]. However, systematic characterization of the *PP2A* family in strawberry has not been reported. The protein phosphatase inhibitor cantharidin, a natural terpenoid from blister beetles, has been widely used as a pharmacological tool to probe PP2A function in plants, yet its application in dissecting ALA-mediated stress signaling remains unexplored. Notably, the PP2A catalytic subunit *MdPP2AC* was recently found to mediate ALA-induced salt tolerance in apple [20]. These findings in apple, together with the known role of PP2A in plant salt tolerance, prompted us to investigate whether PP2A also mediates ALA-induced stress responses in strawberry.

5-Aminolevulinic acid (ALA) is a key precursor in the tetrapyrrole biosynthesis pathway, serving as the universal building block for chlorophyll, heme, siroheme, and phytochromobilin [21]. Beyond its biosynthetic role, ALA has emerged as a multifunctional plant growth regulator capable of enhancing tolerance to diverse abiotic stresses including salinity, drought, heavy metals, and extreme temperatures [22,23]. The physiological effects of exogenous ALA are well-documented across multiple crop species: it promotes photosynthetic capacity, increases antioxidant enzyme activities, reduces reactive oxygen species (ROS) accumulation, and improves ion homeostasis under stress conditions [24,25,26]. However, the molecular mechanisms through which ALA is perceived and transduced to downstream effectors remain poorly defined. Unlike classical hormones, no ALA-specific receptor has been identified [23], and the signaling intermediates linking ALA application to physiological outcomes are only beginning to be elucidated. In strawberry, ALA was demonstrated to enhance salt tolerance through a NO–H_2_O_2_ signaling circuit regulated by the transcription factors FaWRKY70 and FaWRKY40 [27]. Together with the PP2A–ALA functional link established in apple, these findings raise the intriguing possibility that PP2A participates in ALA-mediated salt tolerance in strawberry. Our previous transcriptome analysis revealed that ALA treatment induced the expression of several *PP2A* genes, including both *B56* regulatory subunits and *C2* catalytic subunits, under salt stress, suggesting that ALA may selectively modulate PP2A holoenzyme composition via transcriptional regulation of specific subunits. However, the *PP2A* gene family in strawberry has not been systematically catalogued, the full spectrum of ALA-responsive PP2A members remains unknown, and the functional requirement for PP2A activity in ALA-mediated antioxidant protection has not been experimentally tested.

In this study, we addressed these gaps through three integrated approaches. First, we performed a comprehensive genome-wide identification and evolutionary analysis of the *PP2A* gene family in the octoploid strawberry ‘Benihoppe’, including chromosomal localization, phylogenetic reconstruction, gene duplication patterns, selection pressure analysis, and promoter cis-element characterization. Second, through time-course RNA-seq analysis of root tips under CK, NaCl, and NaCl + ALA treatments across three time points, we systematically identified *PP2A* members responsive to salt stress and ALA treatment and characterized their temporal expression dynamics at subfamily resolution. Third, we employed the protein phosphatase inhibitor cantharidin in physiological experiments to validate the functional requirement of PP2A activity in ALA-mediated antioxidant protection. Our results provide a systematic characterization of the strawberry *PP2A* family, reveal a temporally restricted transcriptional response to ALA under salt stress involving both *B56-η* regulatory and *C2* catalytic subunits, and implicate PP2A as a functional mediator of ALA-induced salt tolerance through pharmacological validation.

## 2. Materials and Methods

### 2.1. Plant Materials and Treatments

Strawberry (*F.* × *ananassa* ‘Benihoppe’) seedlings were cultured hydroponically in ½ Hoagland nutrient solution under controlled conditions (25/20 °C day/night, 16/8 h photoperiod, 60–70% relative humidity). Uniform seedlings at the 4–6 leaf stage were used for all experiments.

For RNA-seq time-course analysis, seedlings were subjected to three treatment groups: (1) CK (½ Hoagland solution), (2) NaCl (200 mM NaCl in ½ Hoagland), (3) NaCl + ALA (200 mM NaCl + 25 mg·L^−1^ ALA in ½ Hoagland). Root tips were harvested at 24 h, 48 h and 72 h after treatment. Three biological replicates were collected for CK and NaCl at 24 h; two to three replicates were used at the other time points. One NaCl + ALA sample at 24 h was excluded after library preparation failed, so the 24 h NaCl + ALA group comprises two biological replicates.

To validate the role of PP2A in ALA-mediated salt tolerance, a separate inhibitor experiment was conducted using cantharidin (CT), a PP2A activity inhibitor, to suppress PP2A activity and verify the enzyme functions. CT was applied at 50 µM, the concentration used to inhibit PP2A activity in *Arabidopsis* seedlings under salt stress [14] and in ALA-related pharmacological studies in apple [20]. Five treatment groups were established: (1) CK, (2) NaCl (200 mM), (3) NaCl + ALA (200 mM NaCl + 25 mg·L^−1^ ALA), (4) NaCl + CT (200 mM NaCl + 50 μM CT), (5) NaCl + ALA + CT (200 mM NaCl + 25 mg·L^−1^ ALA + 50 μM CT). After 4 days of treatment, the third fully expanded leaf from the apex was sampled for physiological and biochemical measurements, with three biological replicates per treatment.

### 2.2. Identification of PP2A Family Members

The strawberry ‘Benihoppe’ reference genome and protein sequences were obtained from the Genome Database for *Rosaceae* (GDR [28]). Arabidopsis thaliana PP2A protein sequences were retrieved from TAIR [29] and used as queries for BLASTP searches [30] (*E*-value < 1 × 10^−5^) against the strawberry proteome. Pfam domain profiles for PP2A subunits were obtained from the Pfam database [31] and used for HMMER [32] searches. Candidate proteins were further validated by checking the presence of characteristic PP2A domains using InterProScan [33] and NCBI CD-Search [34]. Protein sequences with complete PP2A domains and significant BLAST hits were retained as putative PP2A members.

### 2.3. Protein Physicochemical Properties and Subcellular Localization Prediction

The physicochemical properties of the identified FaPP2A proteins, including number of amino acids, molecular weight (MW), theoretical isoelectric point (pI), instability index, aliphatic index, and grand average of hydropathicity (GRAVY), were computed using the ExPASy ProtParam tool (https://web.expasy.org/protparam/ (accessed on 28 July 2026)). Subcellular localization of FaPP2A proteins was predicted using WoLF PSORT [35] with the plant organism type setting.

### 2.4. Phylogenetic Analysis, Gene Structure, and Conserved Motif Identification

Multiple sequence alignment of full-length FaPP2A and AtPP2A protein sequences was performed using MUSCLE (v5.1) [36] implemented in MEGA (v11.0) [37]. A neighbor-joining (NJ) phylogenetic tree was constructed using MEGA with the Poisson correction model and pairwise deletion of gaps. Branch support was assessed using 1000 bootstrap replicates. The resulting tree was visualized and annotated using the Interactive Tree of Life (iTOL) online platform [38]. *FaPP2A* genes were named after their closest *Arabidopsis* homologue, identified by reciprocal BLASTP against the *Arabidopsis PP2A* family. This nomenclature indicates sequence similarity and does not imply functional equivalence.

The exon–intron structure of FaPP2A genes was visualized using TBtools [39] (v2.0) based on the GFF3 annotation file of the ‘Benihoppe’ genome. Conserved motifs in FaPP2A protein sequences were identified using the MEME Suite [40] with the following parameters: maximum number of motifs = 20, minimum motif width = 6, maximum motif width = 50. Conserved domains were further validated using the NCBI Conserved Domain Database (CDD; https://www.ncbi.nlm.nih.gov/cdd/ (accessed on 28 July 2026)) and InterProScan.

### 2.5. Chromosomal Localization and Gene Duplication Analysis

PP2A genes were mapped to strawberry chromosomes based on their genomic coordinates from the GFF annotation file. Gene duplication types were classified as segmental (WGD), tandem, proximal, or dispersed using the MCScanX (v1.1) pipeline [41] with default parameters. Tandem duplications were defined as adjacent homologous genes within 100 kb on the same chromosome. Segmental duplications were identified from syntenic blocks between chromosomes.

### 2.6. Cis-Acting Element Analysis

The 2000 bp upstream promoter sequences of *FaPP2A* genes were extracted from the ‘Benihoppe’ reference genome. Cis-acting regulatory elements were predicted using the PlantCARE database [42]. Identified elements were classified into functional categories (stress-responsive, hormone-responsive, light-responsive, and development-related) and visualized using TBtools.

### 2.7. Ka/Ks Analysis

Coding sequences of duplicated PP2A gene pairs were aligned using MAFFT (v7.5) [43] and converted to codon alignments. Non-synonymous substitution rates (Ka) and synonymous substitution rates (Ks) were calculated using the Nei-Gojobori method [44] implemented in Ka/Ks Calculator [45].

### 2.8. RNA-Seq and Expression Analysis

Total RNA was extracted from root tips using TRIzol reagent. Library preparation and paired-end sequencing (150 bp) were performed on the Illumina NovaSeq 6000 platform by Annoroad Gene Technology (Beijing, China). Reads were aligned to the ‘Benihoppe’ reference genome using HISAT2 (v2.2.1) [46], and gene expression levels were quantified as FPKM (Fragments Per Kilobase per Million mapped reads) using StringTie (v2.2.1) [47]. Expression values were log_2_(FPKM + 1) transformed for downstream analysis. The expression matrix of 75 PP2A genes across all samples (CK, NaCl, and NaCl + ALA at 24 h, 48 h, and 72 h) was extracted. For the heatmap, FPKM values were log2-transformed and row Z-scores were used to display relative expression patterns. Z-scores represent relative patterns and not effect size, and all individual samples of the 24 h time point are shown. For the quadrant analysis, genes were classified by their fold-change direction in response to NaCl versus CK (*x*-axis) and ALA versus NaCl (*y*-axis) at each time point, using a symmetrical |log_2_FC| > 0.2 criterion on both axes. This classification is descriptive and is not used for statistical inference. Differential expression between NaCl and NaCl + ALA at 24 h was tested with DESeq2 (v1.42) on the raw count matrix. Genes with a total count below 5 were filtered out. *p* values were adjusted by the Benjamini–Hochberg procedure, and a gene was considered differentially expressed at FDR < 0.05. For the NaCl versus CK comparison only FPKM values were available; those results are therefore descriptive. Expression heatmaps were generated using the pheatmap package in R (v4.3).

### 2.9. Quantitative Real-Time PCR (qRT-PCR) Validation

Total RNA was extracted from root tips harvested 24 h after treatment using RNA extraction kit (Cat#: RN33050, Biofit, Chengdu, China), and first-strand cDNA was synthesized with reverse transcription kit (One-Step gDNA Removal and cDNA Synthesis Supermix, Transgen, Beijing, China). Gene-specific primers for the six selected FaPP2A genes were designed with Primer3 (v2.6) and are listed in Supplementary Table S1. *FaACTIN* was used as the internal reference. qRT-PCR was performed on a QuantStudio 5 (Applied Biosystems, Waltham, MA, USA) real-time reaction system using a SYBR Green Premix Pro Taq HS qPCR (Accurate Biotechnology Co., Ltd., Changsha, China) Kit (Rox Plus). Relative expression levels were calculated using the 2^−ΔΔCT^ method [48], with three biological replicates per treatment.

### 2.10. Physiological and Biochemical Measurements

The third fully expanded leaf from the apex was used for all physiological and biochemical assays. The contents of O_2_•^−^ and the activities of SOD and CAT, were determined according to Yang et al. [49]. Malondialdehyde (MDA) content was measured following [50]. Proline content was measured by the acid-ninhydrin method [51]. Soluble sugar content was determined by the anthrone colorimetric method [52]. PP2A was determined using a commercial Plant PP2A enzyme-linked immunosorbent assay kit (Shanghai Fusheng Industrial Co., Ltd., Shanghai, China, Item No. A024850-96T) according to the manufacturer’s instructions. The assay is a double-antibody sandwich immunoassay. Samples were bound by a plate-coated purified anti-plant-PP2A antibody and detected with an HRP-conjugated anti-PP2A antibody. Colour was developed with TMB and absorbance was measured at 450 nm. Concentrations were calculated from a standard curve (10–160 IU/L) and corrected for the 5-fold sample dilution; all measured values fell within the standard curve range. The kit uses a polyclonal anti-plant-PP2A antibody pair and does not resolve individual subunits or holoenzymes. The antibody pair has not been independently validated in strawberry.

### 2.11. Determination of Na^+^ and K^+^ Contents

Leaf and root samples were harvested after 4 days of treatment, rinsed three times with deionised water, blotted dry, and oven-dried at 80 °C to constant weight. Dried tissue was ground to a fine powder and digested in concentrated nitric acid. Na^+^ and K^+^ concentrations were determined by inductively coupled plasma optical emission spectrometry (ICP-OES) and are expressed as mg g^−1^ dry weight. The Na^+^/K^+^ ratio was calculated as the mass ratio of the two ions. Data are presented as mean ± SEM of three biological replicates.

### 2.12. Statistical Analysis

All experiments were performed with three biological replicates unless otherwise stated. The 24 h NaCl + ALA RNA-seq group comprised two biological replicates (Section 2.1). RNA-seq differential expression was tested with DESeq2 (Section 2.8). Data are presented as mean ± SEM. Statistical significance was determined by one-way ANOVA followed by Tukey’s HSD post hoc test for multiple comparisons, or Student’s *t*-test for pairwise comparisons. For multiple comparisons, different lowercase letters indicate significant differences at *p* < 0.05 by Tukey’s HSD test. For pairwise comparisons, significance was determined at *p* < 0.05 by Student’s *t*-test.

## 3. Results

### 3.1. Identification of 75 PP2A Genes in Strawberry

To comprehensively identify PP2A family members in strawberry, we performed BLASTP and HMMER searches using Arabidopsis PP2A proteins and Pfam domain profiles as queries against the ‘Benihoppe’ proteome. After removing redundant sequences and verifying domain integrity, a total of 75 *PP2A* genes were identified. These were classified into five subfamilies based on sequence homology and domain architecture: *A* (scaffold subunit, 2 genes), *B55* (regulatory subunit, 10 genes), *B56* (regulatory subunit, 29 genes), *B″* (regulatory subunit, 14 genes), and *C* (catalytic subunit, 20 genes) (Supplementary Table S2). Notably, the regulatory B subunits accounted for 70.7% (53/75) of the entire PP2A family, with B56 being the largest subfamily (38.7%), reflecting the regulatory diversity required for substrate-specific dephosphorylation in strawberry. The 75 PP2A members were named after their closest *Arabidopsis* homologue, identified by reciprocal BLASTP (Supplementary Table S2). Names indicate sequence similarity and do not imply functional equivalence.

**Figure 1:**
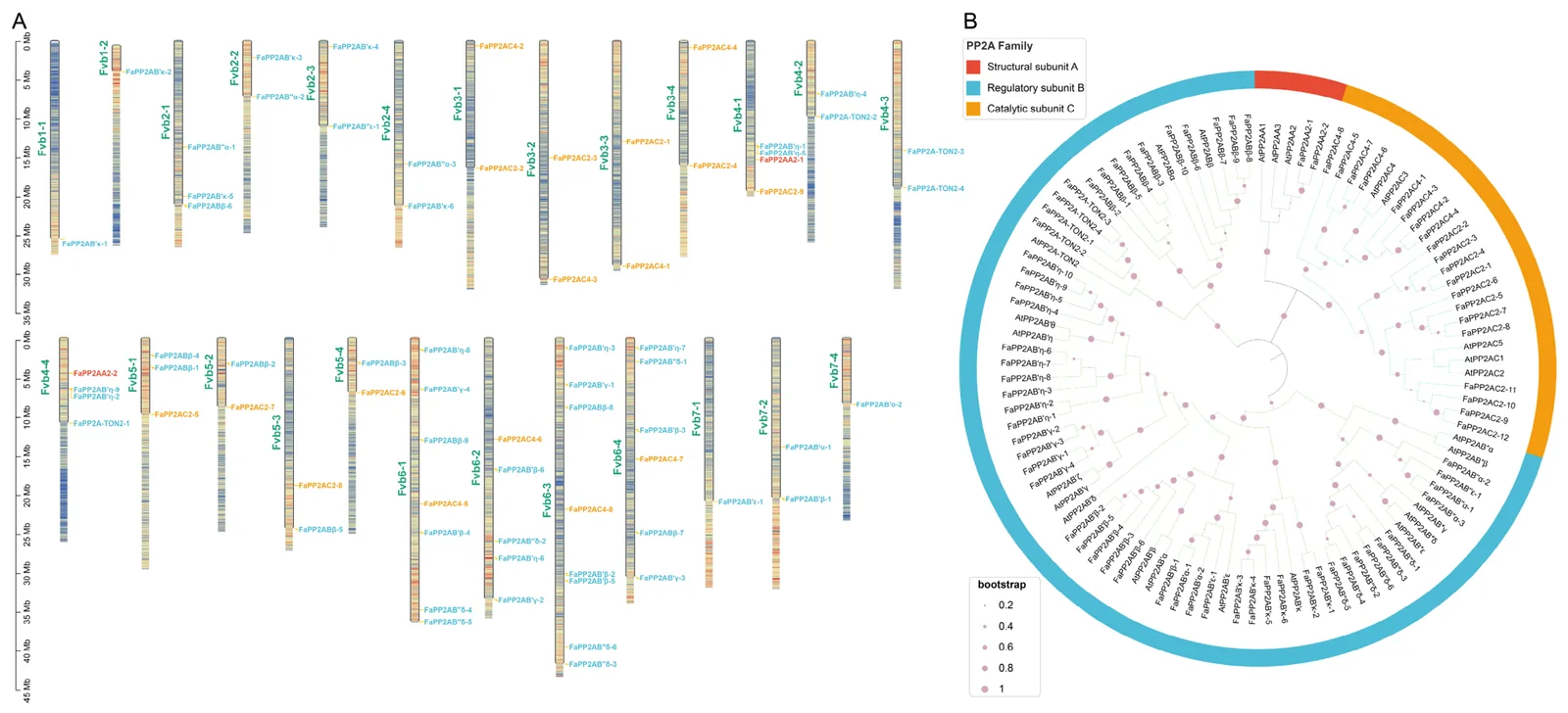
Genome-wide characterization of the *PP2A* gene family in strawberry. (**A**) Chromosomal distribution of the 75 *FaPP2A* genes across the octoploid strawberry (*F*. × *ananassa* ‘Benihoppe’) genome. Chromosome numbers are indicated at the top. Sub-genomes are distinguished by color. Chromosome coloring from light to dark indicates the density of *FaPP2A* genes (number of genes per 1-Mb window) (**B**) Phylogenetic tree of PP2A proteins from strawberry (*F*. × *ananassa*) and *Arabidopsis thaliana*. The neighbor-joining tree was constructed using MEGA v11.0 with the Poisson correction model and 1000 boot-strap replicates. A subunits are shown in red, B subunits in blue, and C subunits in yellow. Bootstrap support values ≥ 50% are shown at nodes.

The 75 *FaPP2A* genes were unevenly distributed across chromosomes, with subgenome-specific patterns characteristic of the octoploid strawberry genome. Phylogenetic analysis resolved the five subfamilies into distinct clades with strong bootstrap support (Figure 1B). The *A* and *C* subfamilies formed monophyletic groups with high sequence conservation, while *B* subfamilies showed greater sequence divergence. Together, the chromosomal distribution and phylogenetic reconstruction provide a genomic and evolutionary framework for the strawberry PP2A family (Figure 1).

### 3.2. Physicochemical Properties and Subcellular Localization of FaPP2A Proteins

Physicochemical analysis revealed subfamily-specific profiles that mirror the functional roles of PP2A subunits (Supplementary Table S3). *C* catalytic subunits were the smallest and most stable, with acidic isoelectric points, consistent with their conserved enzymatic function [15]. A scaffold subunits were uniquely hydrophobic (positive GRAVY) and exhibited the highest aliphatic index, reflecting the HEAT-repeat scaffold structure that mediates B and C subunit binding. Among *B* regulatory subunits, B55 members displayed the greatest structural diversity and hydrophilicity; B56 uniquely contained basic isoforms (20 of 29 B56 proteins, comprising all basic members of the family), a feature that may facilitate electrostatic substrate interactions; and B″ members, including TON2 homologs, showed the narrowest pI range, consistent with specialized roles in cytoskeletal organization. The predominance of predicted unstable proteins in B55 and B56 subfamilies suggests that regulatory subunits may undergo rapid turnover.

Subcellular localization prediction revealed a nuclear (45.3%)–cytoplasmic (41.3%) dual distribution for FaPP2A proteins, with minor predicted targeting to mitochondria, chloroplast, endoplasmic reticulum, plasma membrane, and cytoskeleton (Supplementary Table S3). A subunits were predicted to localize to the ER, supporting the hypothesis that holoenzyme assembly initiates at the ER membrane, while C subunits were predicted to be predominantly cytoplasmic. Among B subunits, B55 and B″ were predicted to be largely nuclear, whereas B56 showed a predicted mixed distribution, reflecting functional diversification within this largest subfamily.

### 3.3. Gene Structure and Conserved Domain Architecture

**Figure 2:**
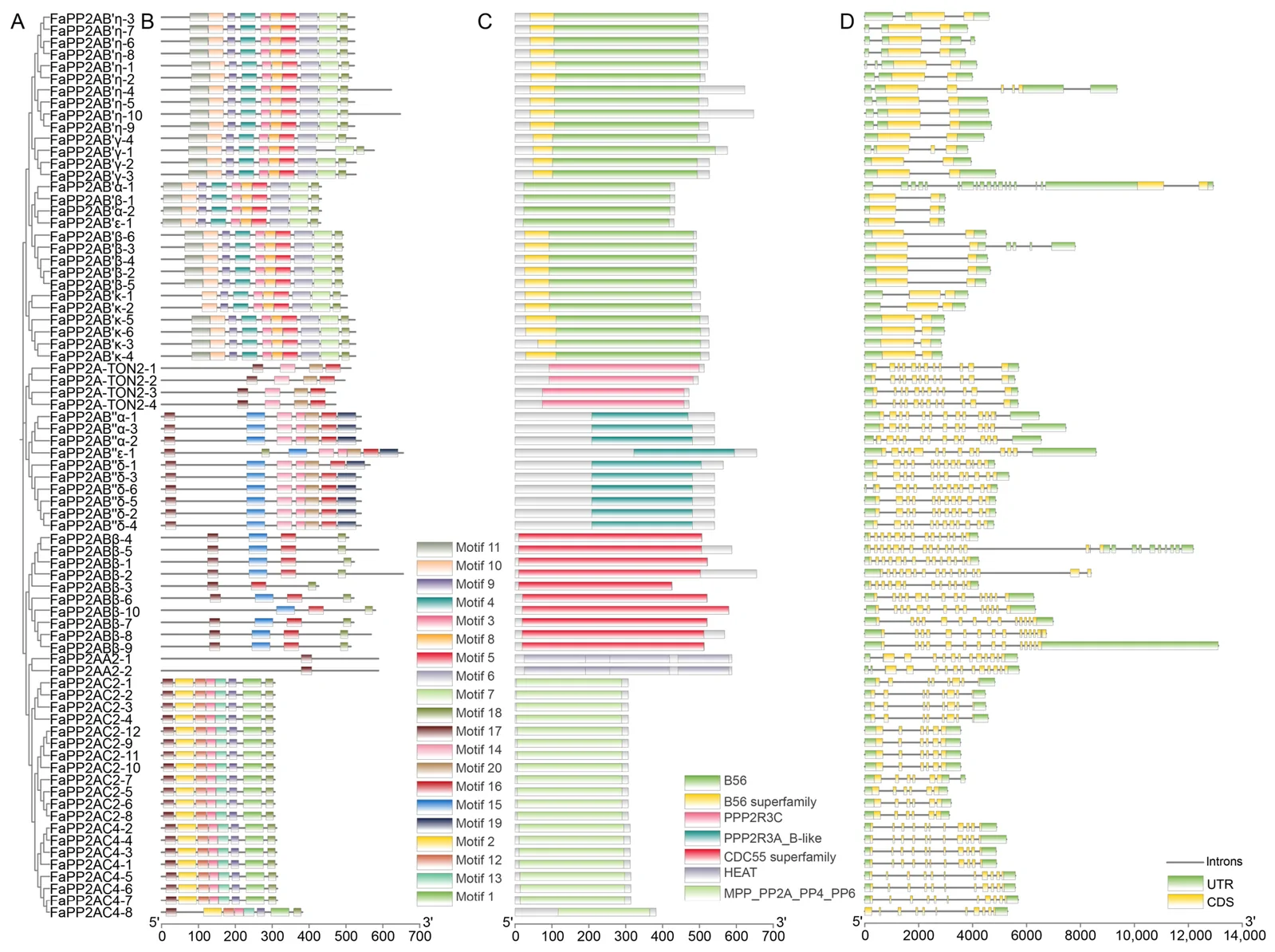
Phylogenetic relationships and conserved architecture of the 75 FaPP2A proteins. (**A**) Phylogenetic tree constructed using the neighbor-joining method with 1000 bootstrap replicates. (**B**) Conserved motif distribution identified by MEME Suite. Twenty motifs are represented by differently colored boxes and are arranged along each protein sequence according to their positions. (**C**) Conserved domain (CDD) analysis. (**D**) Exon–intron structure. Yellow boxes represent CDS, green boxes represent UTR, and black lines represent introns. Gene lengths and exon numbers are drawn to scale.

To characterize the conserved sequence features of FaPP2A proteins, MEME analysis identified 20 conserved motifs across the 75 proteins (Figure 2B). Several motifs were widely distributed across all subfamilies, while others showed subfamily-specific enrichment patterns. Motifs corresponding to the PP2Ac phosphatase catalytic core were present in all C subunits, and HEAT-repeat-associated motifs were characteristic of A scaffold subunits. Among B regulatory subunits, B55 and B″ members shared a subset of motifs distinct from those enriched in B56 members, consistent with their phylogenetic separation. Conserved Domain Database (CDD) searches further validated these findings (Figure 2C): the PP2Ac phosphatase domain (PF00149) was universally detected in C subunits, HEAT repeat domains were specific to A subunits, the B56 domain (PF01603) was restricted to B56 members, and the B55 domain (PF03737) was confined to B55 members. These domain assignments were fully concordant with the subfamily classification based on phylogenetic analysis. Gene structure analysis revealed that the number of CDS (exons) in *FaPP2A* genes ranged from 2 to 17, indicating substantial variation in gene architecture (Figure 2D). Notably, *B56* subfamily genes possessed the fewest exons, averaging approximately 2–5, whereas *B55* members contained the most complex gene structures, averaging 12–17 exons. *A* and *B″* subunit genes exhibited intermediate exon counts. Within each subfamily, exon–intron organization was highly conserved, consistent with the phylogenetic clustering and conserved motif patterns described above.

### 3.4. Cross-Species Sequence Conservation of PP2A Proteins

**Figure 3:**
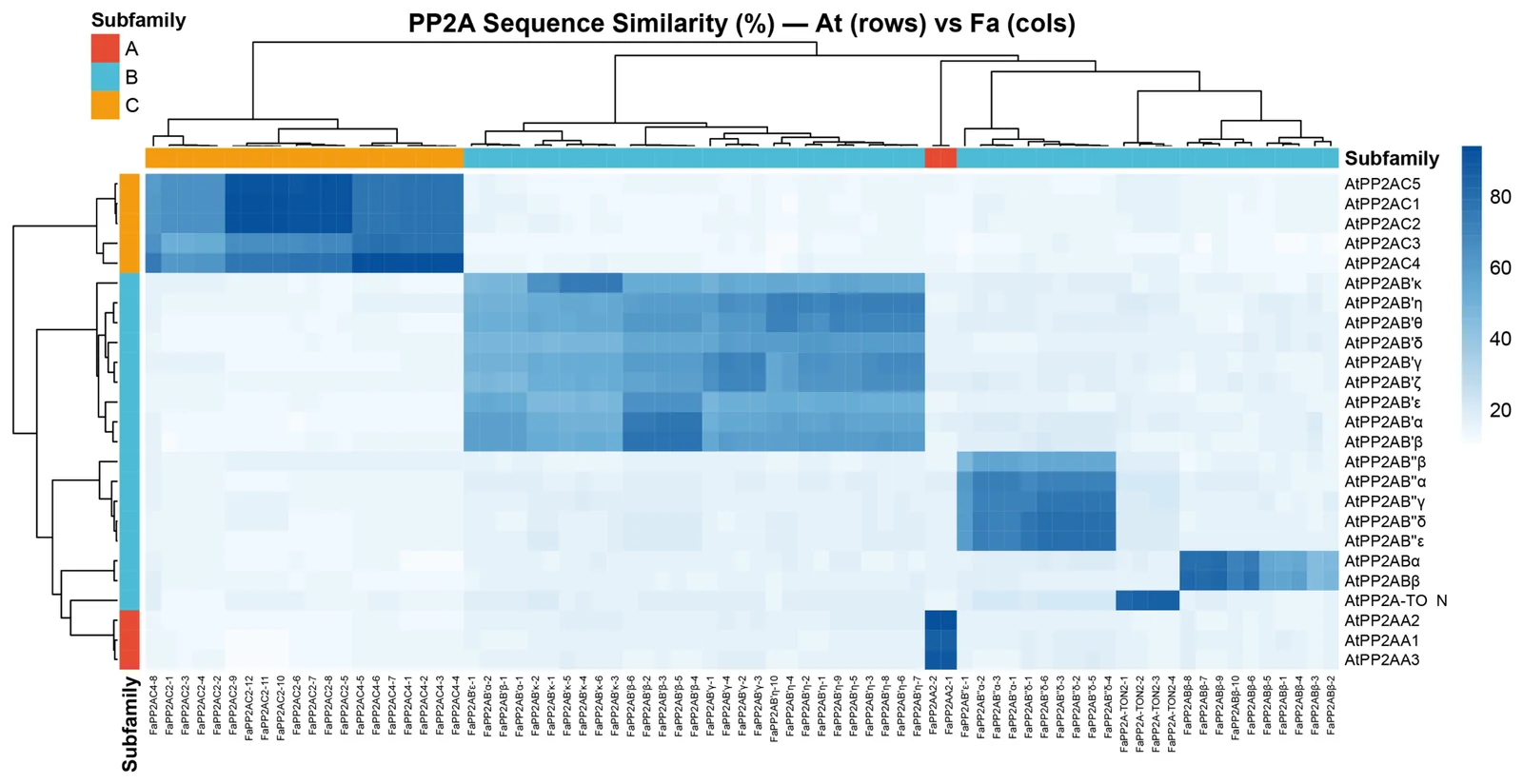
Sequence similarity matrix between strawberry (FaPP2A) and *Arabidopsis* (AtPP2A) PP2A proteins. Color intensity represents percentage sequence identity, with darker colors indicating higher similarity. The left color bar indicates *Arabidopsis* subfamily classification, and the top color bar indicates strawberry subfamily classification. All FaPP2A proteins showed high sequence identity (>50%) with their corresponding closest *AtPP2A* homologues within the same subfamily, with clear subfamily-level boundaries. FaPP2AA and FaPP2AC subunits exhibited the highest cross-species similarity (>85%), while FaPP2AB regulatory subunits displayed a wider similarity range (50–85%), consistent with the functional diversification of regulatory subunits.

To support the nomenclature based on sequence similarity, we compared all FaPP2A and AtPP2A proteins and visualized the pairwise identities as a similarity matrix (Figure 3). All FaPP2A members showed high sequence identity (>50%) with their closest *Arabidopsis* homologue within the same subfamily, with clear subfamily-level boundaries. FaPP2AA and FaPP2AC subunits exhibited the highest cross-species similarity (>85%), consistent with their conserved structural and catalytic functions, while FaPP2AB regulatory subunits displayed a wider similarity range (50–85%), reflecting their functional diversification. This result further supports the reliability of the phylogeny-based subfamily classification.

### 3.5. Cis-Acting Element Analysis of FaPP2A Promoters

**Figure 4:**
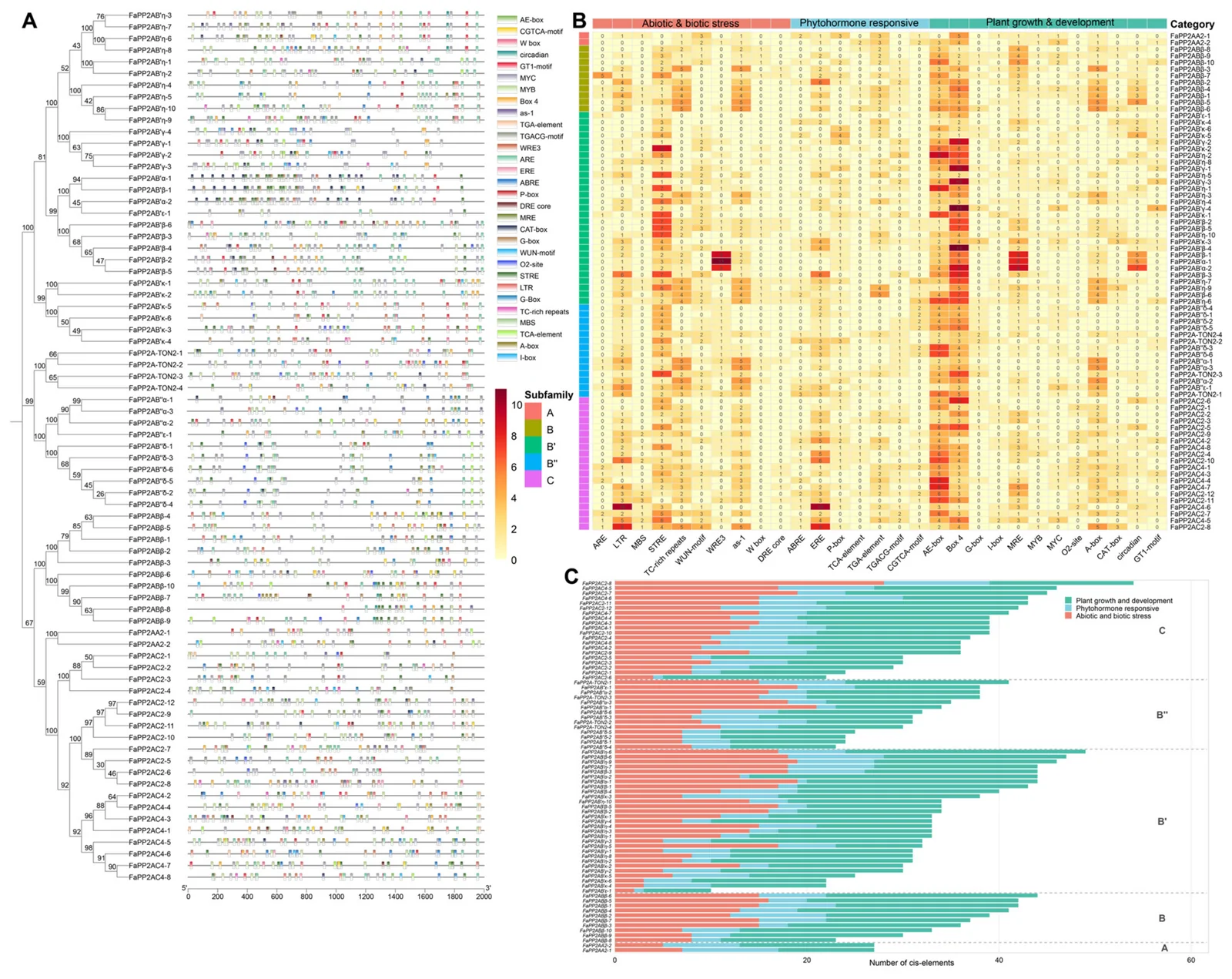
Cis-acting regulatory element analysis of *FaPP2A* gene promoters. (**A**) Distribution of 2617 cis-acting elements identified in the 2000 bp upstream promoter regions of 75 *FaPP2A* genes, classified into three major categories: abiotic/biotic stress-responsive, phytohormone-responsive, and plant growth/development-related. (**B**) Heatmap showing the abundance and distribution of individual cis-element types across *FaPP2A* promoters grouped by subfamily. The color scale represents element copy number. (**C**) Stacked bar chart of the number of cis-acting elements per functional category in each FaPP2A promoter, with genes grouped by subfamily. Green, light blue, and red segments represent plant growth/development-related, phytohormone-responsive, and abiotic/biotic stress-responsive elements, respectively; the x-axis shows the number of cis-acting elements.

To gain insight into the potential regulatory mechanisms of *PP2A* genes, we analyzed the 2000 bp upstream promoter sequences of all 75 *FaPP2A* members using PlantCARE. A total of 2617 cis-acting regulatory elements were identified and classified into three major categories: abiotic/biotic stress-responsive (34.3%), phytohormone-responsive (17.2%), and plant growth/development-related (48.6%) (Figure 4). *B56* subfamily promoters were enriched in stress- and hormone-responsive elements, consistent with their dynamic transcriptional response to NaCl and ALA treatment. In contrast, *C2* catalytic subunit promoters contained fewer stress-responsive elements, in line with their relatively stable expression across treatments, while *C4* promoters lacked several hormone-responsive motifs, which may contribute to their transcriptional suppression under salt stress. MYB and MYC transcription factor binding sites, known regulators of stress-responsive gene expression, were identified in promoters across all subfamilies. The diversity of cis-acting elements, with subfamily-specific enrichment patterns, prompted us to examine the evolutionary forces and expression dynamics of these genes.

### 3.6. Selection Pressure and Duplication Patterns

**Figure 5:**
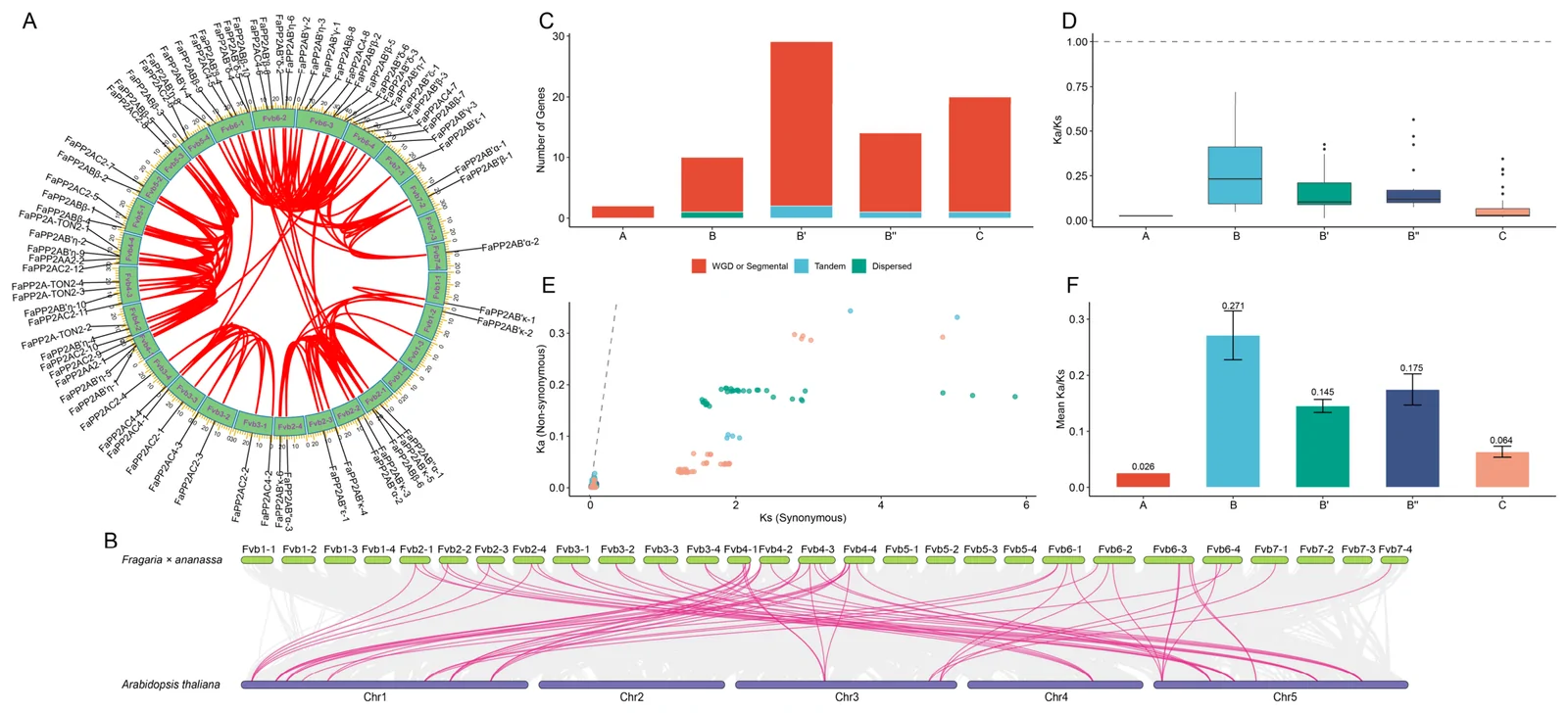
Gene duplication and selection pressure analysis of *FaPP2A* genes. (**A**) Intraspecific collinearity of duplicated *FaPP2A* gene pairs across the octoploid strawberry genome. Red lines connect syntenic FaPP2A gene pairs. (**B**) Interspecific collinearity between strawberry and *Arabidopsis;* pink lines connect orthologous gene pairs. (**C**) Distribution of duplication types by subfamily. Segmental duplication (WGD/Segmental, blue) dominates (93%), with tandem duplication (orange) occurring in B56, B″, and C and dispersed duplication (light blue) occurring only once in B55. (**D**) Boxplot of Ka/Ks ratios. Dashed line indicates Ka/Ks = 1 (neutral selection). All subfamily medians are well below 1, indicating strong purifying selection; C subfamily shows the narrowest distribution. (**E**) Ka/Ks scatter plot. Each point represents one duplicated gene pair, colored by duplication type: blue, orange, and light blue indicate WGD/segmental, tandem, and dispersed duplications, respectively. The diagonal dashed line indicates Ka = Ks. All points fall below the diagonal, confirming pervasive purifying selection. (**F**) Mean Ka/Ks SE by subfamily. C subunits (Ka/Ks = 0.064) are the most conserved, while B55 subunits (Ka/Ks = 0.271) show the greatest sequence plasticity.

Intraspecific collinearity analysis revealed extensive segmental duplication of *FaPP2A* genes across the octoploid strawberry genome (Figure 5A), while interspecific collinearity with *Arabidopsis* was consistent with the subfamily classification (Figure 5B). Gene duplication analysis showed that segmental duplication (WGD) was the predominant mechanism for PP2A family expansion, accounting for 93.3% of members, with tandem duplication contributing 5.3% and dispersed duplication only 1.3% (Figure 5C). This predominance of segmental duplication is consistent with the demonstration that whole-genome duplications drove the expansion of most *PP2A* subunit gene families in flowering plants [18]. Tandem duplications were found in the *B56*, *B″*, and *C* subfamilies, and a single dispersed duplication event was detected in B55.

To assess the evolutionary forces shaping the PP2A family, we calculated Ka/Ks ratios for 171 duplicated gene pairs. All subfamily medians fell well below 1, confirming pervasive purifying selection throughout the evolutionary history of the strawberry PP2A family (Figure 5D). The Ka/Ks scatter plot showed all gene pairs below the neutral diagonal, further supporting the dominance of purifying selection (Figure 5E). The mean Ka/Ks values differed significantly among subfamilies (Figure 5F): C subunits (Ka/Ks = 0.064) were the most conserved, consistent with their essential catalytic function, while B55 subunits (Ka/Ks = 0.271) exhibited the greatest sequence plasticity, suggesting relaxed selective constraints that may facilitate regulatory diversification and neofunctionalization of substrate-specific adaptors. The mean Ka/Ks for A (0.026), B56 (0.145), and B″ (0.175) subfamilies fell between these extremes.

### 3.7. Transcriptome Analysis Identifies PP2A Members Responsive to Salt and ALA

**Figure 6:**
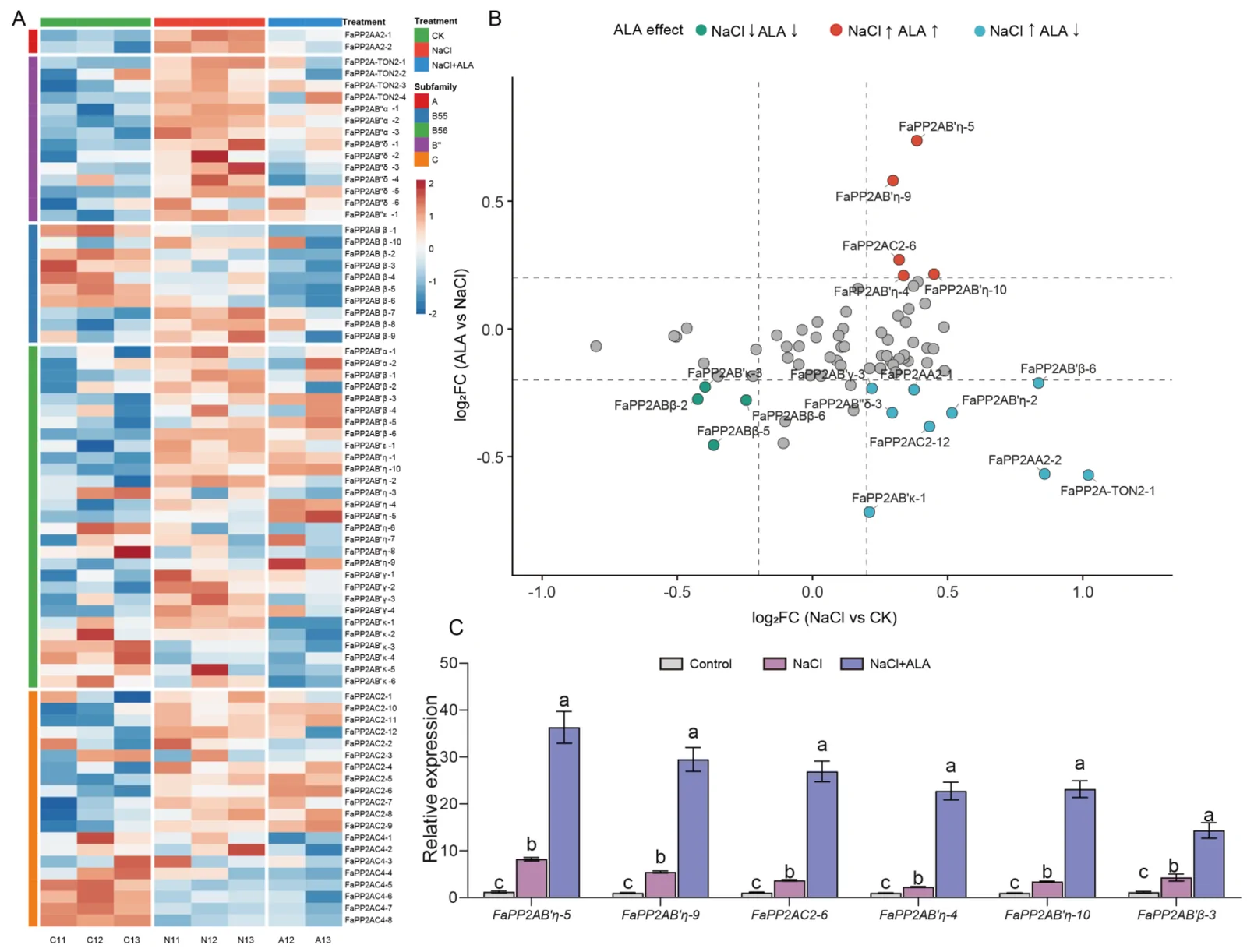
Transcriptional response of the 75 *FaPP2A* genes under CK, NaCl, and NaCl + ALA treatments at 24 h. (**A**) Sample-level expression heatmap. Columns represent the eight individual samples of the 24 h time point (CK, three replicates; NaCl, three replicates; NaCl + ALA, two replicates). FPKM values were log_2_-transformed and row Z-scored; Z-scores represent relative patterns and not effect size. (**B**) Four-quadrant scatter plot of ALA effect. Red (*n* = 5): NaCl-up, ALA-enhanced, predominantly four B56-η members and *FaPP2AC2-6*. Blue (*n* = 9): NaCl-up, ALA-reversed. Green (*n* = 4): NaCl-down, further suppressed by ALA (three B55 and *FaPP2AB′κ-3*). Gray (*n* = 57): below threshold. Gene names are labeled for colored quadrants. This classification is descriptive and is not used for statistical inference. (**C**) qRT-PCR validation of the six most strongly ALA-enhanced genes (*FaPP2AB′η-5*, *FaPP2AB′η-9*, *FaPP2AC2-6*, *FaPP2AB′η-10*, *FaPP2AB′η-4*, and *FaPP2AB′β-3*) under CK, NaCl, and NaCl + ALA treatments at 24 h, bars show mean ± SEM of three biological replicates. Different lowercase letters above bars indicate significant differences among treatments at *p* < 0.05 by Tukey’s HSD test.

To investigate the transcriptional response of *PP2A* genes to salt stress and ALA treatment, we performed RNA-seq on root tips under CK, NaCl, and NaCl + ALA at 24 h (three biological replicates for CK and NaCl and two for NaCl + ALA; Figure 6A). All 75 PP2A members were expressed in at least one condition, with the majority exhibiting moderate expression changes. Expression patterns differed between subfamilies: B″ members showed the most pronounced salt-responsive upregulation, whereas several C4 catalytic subunits were markedly downregulated by NaCl, and B55 members were suppressed under both NaCl and ALA. In contrast, C2 members were generally stable or mildly upregulated by NaCl, suggesting that different PP2A subunits play distinct, possibly antagonistic, roles in the salt stress response.

To identify ALA-enhanced genes, we constructed a four-quadrant scatter plot (Figure 6B). DESeq2 identified three *PP2A* genes as differentially expressed between NaCl and NaCl + ALA at 24 h (FDR < 0.05), all from the B56 subfamily: *FaPP2AB′η-5*, *FaPP2AB′η-9* and *FaPP2AB′κ-1*. Six further genes reached nominal *p* < 0.05. In the descriptive quadrant plot, five genes fell into the NaCl-up, ALA-enhanced quadrant (four B56-η members and *FaPP2AC2-6*), nine were NaCl-up and ALA-reversed, and four were NaCl-down and further suppressed; the remaining 57 genes fell below the |log_2_FC| > 0.2 threshold. Temporal analysis revealed that the ALA-enhanced response was transient: five genes fell in the ALA-enhanced quadrant at 24 h, none at 48 h and one at 72 h, with responsive B56 subclasses shifting from B56-η at 24 h to B56-κ at later stages. Together, these results reveal a subfamily-specific transcriptional hierarchy in which ALA predominantly enhances *B56-η* regulatory subunits, differentially modulates *C2* isoforms, and suppresses *B55* and *C4* members, with this regulation being most pronounced during early stress adaptation. The dependence of this descriptive classification on the threshold is summarised in Table S4.

Among the genes identified by DESeq2 and the descriptive quadrant analysis, the six with the largest ALA effect were selected for independent qRT-PCR validation: *FaPP2AB′η-5*, *FaPP2AB′η-9*, *FaPP2AC2-6*, *FaPP2AB′η-10*, *FaPP2AB′η-4* and *FaPP2AB′β-3* (Figure 6C). The two datasets differ in replication: the qRT-PCR used three biological replicates per treatment, each measured in technical triplicate, whereas the RNA-seq comparison at 24 h used three biological replicates for CK and NaCl and two for NaCl + ALA. In the qRT-PCR analysis all six genes increased significantly from NaCl to NaCl + ALA (*p* < 0.05), and all six showed the same CK < NaCl < NaCl + ALA direction in both datasets. Five of the six belong to the B56 subfamily (η and β); the remaining gene, FaPP2AC2-6, belongs to the C2 catalytic subclass, indicating that the ALA-responsive increase is not restricted to B regulatory subunits.

### 3.8. ALA Enhances Antioxidant Enzyme Activities and Reduces Oxidative Damage Under Salt Stress

To determine whether the PP2A transcriptional changes observed by RNA-seq translate to functional consequences at the physiological level, we measured a suite of growth, oxidative stress, antioxidant, and osmotic adjustment parameters in strawberry seedlings under five treatments (CK, NaCl, NaCl + ALA, NaCl + CT, and NaCl + ALA + CT) for 4 days.

**Figure 7:**
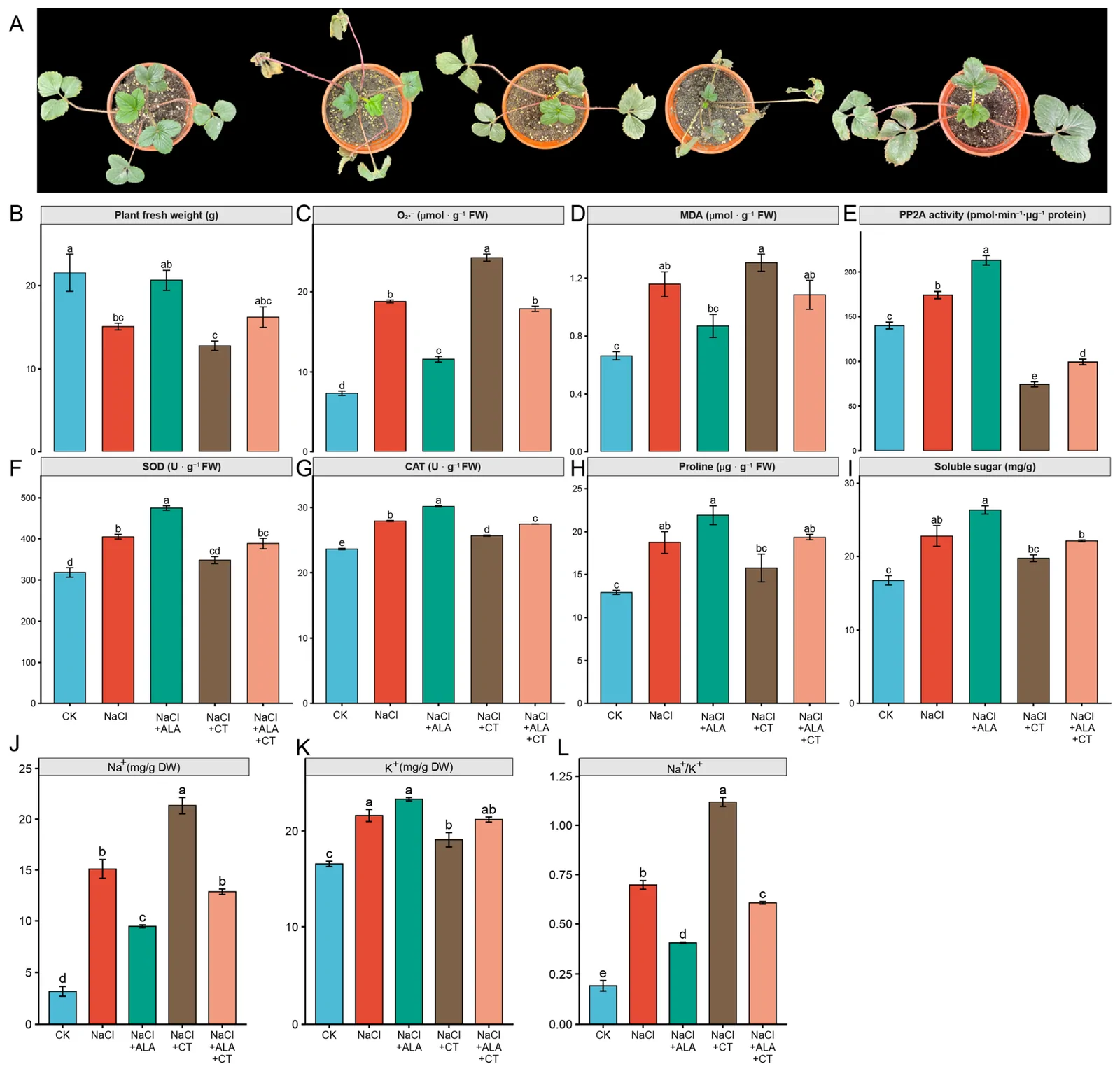
Effects of 5-aminolevulinic acid (ALA) and cantharidin (CT) on strawberry seedlings under NaCl stress after 4 days of treatment. (**A**) Representative phenotype of seedlings from each treatment group (CK, NaCl, NaCl + ALA, NaCl + CT, NaCl + ALA + CT). (**B**) Plant fresh weight. (**C**) O_2_•^−^ production rate. (**D**) MDA content. (**E**) PP2A activity (pmol·min^−1^·µg^−1^ protein). (**F**) SOD activity. (**G**) CAT activity. (**H**) Proline content. (**I**) Soluble sugar content. (**J**) Leaf Na^+^ content. (**K**) Leaf K^+^ content. (**L**) Leaf Na^+^/K^+^ ratio. Data in (**B**–**L**) are presented as mean ± SEM (*n* = 3). Different lowercase letters above bars indicate significant differences among treatments at *p* < 0.05 by Tukey’s HSD test.

NaCl stress significantly reduced plant fresh weight by 30.1% compared with CK, whereas exogenous ALA application (NaCl + ALA) restored plant fresh weight to a level not significantly different from CK (Figure 7B). In contrast, treatment with the PP2A inhibitor cantharidin in the absence of ALA (NaCl + CT) further reduced plant fresh weight to 59.3% of CK, and co-treatment with ALA and CT (NaCl + ALA + CT) resulted in a 21.7% decrease relative to the NaCl + ALA treatment, indicating that CT partially blocked the growth-restoring effect of ALA. Consistent with this pattern, NaCl stress markedly elevated leaf O_2_•^−^ production rate and MDA content by 156.1% and 74.7%, respectively, compared with CK (Figure 7C,D). ALA treatment significantly reduced both O_2_•^−^ and MDA levels, whereas the NaCl + CT group exhibited the highest oxidative damage among all treatments, significantly exceeding even the NaCl group. The alleviation of oxidative stress by ALA was largely attenuated by CT co-treatment, with O2•^−^ and MDA levels in the NaCl + ALA + CT group significantly exceeding those in the NaCl + ALA group.

Total PP2A was measured by immunoassay (Figure 7E). NaCl stress significantly increased PP2A relative to CK, and ALA co-treatment further elevated it to the highest level among all groups. By contrast, cantharidin (CT) reduced PP2A to the lowest level, significantly below CK, and CT co-treatment largely attenuated the ALA-induced increase, with PP2A in the NaCl + ALA + CT group remaining significantly below that in the NaCl + ALA group. The reduction in the CT-treated groups should be interpreted with caution, because the assay reports total PP2A and cantharidin also inhibits protein phosphatase 1.

Consistent with this PP2A activation profile, parallel trends were observed for antioxidant enzyme activities. NaCl stress induced 27.4% and 18.2% increases in SOD and CAT activities relative to CK, respectively (Figure 7F,G), reflecting a basal antioxidant response. ALA treatment further elevated both enzyme activities to the highest levels among all groups, whereas enzyme activities in the NaCl + CT group declined to levels comparable to CK, significantly below those in the NaCl group. SOD and CAT activities in the NaCl + ALA + CT group were significantly lower than those in the NaCl + ALA group, indicating that ALA-mediated activation of antioxidant enzymes requires PP2A activity. Similarly, NaCl stress increased leaf proline and soluble sugar contents by 45.0% and 35.7%, respectively (Figure 7H,I), and ALA further promoted osmolyte accumulation, an effect attenuated by CT.

To assess ion homeostasis directly, we measured Na^+^ and K^+^ contents in leaves and roots (Figure 7J–L and Figure S2). NaCl stress markedly increased leaf Na^+^ and raised the leaf Na^+^/K^+^ ratio (Figure 7J,L). ALA significantly alleviated this, reducing both leaf Na^+^ content and the leaf Na^+^/K^+^ ratio relative to NaCl alone. Leaf K^+^ tended to be higher in the NaCl + ALA group than in the NaCl group, but the difference was not significant (Figure 7K). In roots, ALA had a comparable effect: root Na^+^ accumulation decreased, root K^+^ content increased and the Na^+^/K^+^ ratio decreased relative to NaCl alone (Figure S2). Co-application of cantharidin with ALA significantly reversed these effects in both tissues. The Na^+^/K^+^ ratio in the NaCl + ALA + CT group was higher than in the NaCl + ALA group in both leaves and roots (Figure 7L and Figure S2). The ion data are therefore consistent with the growth, antioxidant and oxidative-damage parameters, and support a contribution of PP2A activity to the ALA response under salt stress.

Taken together, these results indicate that ALA alleviates NaCl stress by mitigating oxidative damage, enhancing antioxidant enzyme activities, promoting osmolyte accumulation and improving leaf and root ion homeostasis. The PP2A inhibitor CT significantly reversed these protective effects, including the reduction in the Na^+^/K^+^ ratio, suggesting that PP2A activity contributes to the ALA-mediated salt stress tolerance pathway in strawberry.

## 4. Discussion

### 4.1. Expansion and Evolution of the Strawberry PP2A Family

The identification of 75 *PP2A* genes in the octoploid strawberry genome represents a substantial expansion compared to diploid model plants such as *Arabidopsis* (26 PP2A subunits) [10] and rice (30 members) [53]. This expansion is primarily driven by segmental duplication (93.3% of members), consistent with the polyploid origin of the cultivated strawberry, which arose through hybridization of four diploid progenitor species [4]. All duplicated pairs exhibited Ka/Ks < 1 (mean Ka = 0.060, mean Ks = 0.940, mean Ka/Ks = 0.138), indicating pervasive purifying selection. This result is consistent with the classical model of gene duplication fate, in which dosage balance and subfunctionalization preserve duplicated genes [54], and mirrors the finding that whole-genome duplications drove the expansion of most *PP2A* subunit gene families in flowering plants [16,17,18]. Notably, Booker and DeLong [18] also showed that the expansion of the *B56* (*PPP2R5*) family was driven by functional diversification rather than by maintenance of gene dosage, and that functionally specialized subclades can revert to singleton status after duplication—a pattern broadly consistent with the pronounced expansion of the *B56* subfamily (29 members, 38.7%) and the restricted occurrence of tandem duplication (5.3%) in the octoploid strawberry *PP2A* repertoire. The relatively high Ks values (mean 0.940) suggest that the duplication events are ancient, likely coinciding with the polyploidization events that gave rise to the octoploid strawberry genome. Notably, tandem duplication was restricted to *B* regulatory and *C* catalytic subfamilies, with only 4 genes (5.3%) arising from this mechanism, further underscoring the dominant role of whole-genome duplication in shaping the octoploid strawberry *PP2A* repertoire.

The *B56* subfamily exhibited the most pronounced expansion (29 members, 38.7% of the family), consistent with recent findings in tea [15] and rubber tree [16], and with the proposal that B56 expansion in plants was driven by functional diversification [18]. Cross-species protein sequence comparison with *Arabidopsis* was consistent with the nomenclature based on sequence similarity, with scaffold (A) and catalytic (C) subunits showing >85% identity to their *Arabidopsis* counterparts, while *B* regulatory subunits displayed a wider similarity range (50–85%), reflecting their functional diversification. The relatively higher Ka/Ks values in B regulatory subfamilies (B55: 0.271, B″: 0.175, B56: 0.145) compared to the catalytic C (0.064) and scaffold A (0.026) subunits support the model that regulatory subunit diversification provides substrate specificity while catalytic functions remain highly constrained [11].

### 4.2. Physicochemical and Subcellular Profiles Reflect Functional Specialization of PP2A Subunits

The subfamily-specific physicochemical profiles of FaPP2A proteins closely mirror their functional roles within the holoenzyme. C catalytic subunits are the smallest and most stable FaPP2A proteins, with acidic isoelectric points, features conserved across eukaryotes that reflect the structural constraints required for maintaining phosphatase activity [9,15]. A scaffold subunits are uniquely hydrophobic (positive GRAVY), consistent with the hydrophobic HEAT-repeat platform that mediates B and C subunit binding [9]. Among regulatory subunits, B55 members display the greatest hydrophilicity and structural diversity, B56 uniquely contains basic isoforms that may facilitate electrostatic substrate interactions, and B″ (including TON2 homologs) shows the narrowest pI range, likely related to specialized roles in cytoskeletal organization [55]. The predominance of predicted unstable proteins among B55 (90%) and B56 (62%) subfamilies suggests that regulatory subunits undergo rapid turnover, enabling dynamic remodeling of PP2A holoenzyme composition in response to environmental signals.

Subcellular localization prediction revealed a nuclear–cytoplasmic dual distribution for the majority of FaPP2A proteins, consistent with their roles in both transcriptional regulation and cytoplasmic signaling [11]. A subunits were uniquely predicted to localize to the ER, supporting the hypothesis that PP2A holoenzyme assembly initiates at the ER membrane [9], while C catalytic subunits were predicted to be predominantly cytoplasmic. Among B subunits, B55 and B″ members were predicted to be largely nuclear, whereas B56 showed a predicted mixed distribution, reflecting functional diversification. The minor predicted organellar localizations (mitochondria, chloroplast) suggest potential roles for PP2A in organellar signaling that remain to be explored.

### 4.3. Promoter Cis-Elements Suggest Multifaceted Regulation of PP2A Genes

Promoter analysis identified a rich repertoire of cis-acting regulatory elements in *FaPP2A* genes in silico, with a predominance of light-responsive (48.6%) and stress-responsive (34.3%) elements. The high abundance of STRE elements is particularly noteworthy, as STRE elements mediate transcriptional responses to a broad spectrum of abiotic stresses. Multiple hormone-responsive elements, including ERE (ethylene) and TGA-element (auxin), were identified in FaPP2A promoters. Whether these elements mediate phytohormone-dependent regulation of FaPP2A expression remains to be tested.

The enrichment of stress- and hormone-responsive elements in *B56* regulatory subunit promoters is consistent with their transcriptional responsiveness to NaCl and ALA treatment observed in our RNA-seq data. In contrast, the *C2* and *C4* catalytic subunit promoters showed distinct element profiles, aligning with their divergent expression patterns under salt stress. MYB and MYC binding sites were identified across multiple subfamilies, which raises the possibility that stress-activated transcription factors contribute to PP2A transcriptional regulation.

### 4.4. Temporal Specificity of ALA-Mediated PP2A Regulation

Our RNA-seq time-course analysis across 24 h, 48 h, and 72 h revealed a striking temporal specificity in ALA’s regulatory effects on *PP2A* expression. The number of ALA-enhanced PP2A genes under salt stress decreased sharply from 5 at 24 h to only 1 at 72 h, with no gene consistently responsive across all time points. This temporal window aligns with the broader transcriptomic response to salt stress, where the total number of differentially expressed genes declined from 9,576 at 24 h to 745 at 72 h, consistent with previous reports that early transcriptional responses dominate salt stress adaptation [5].

The temporal specificity was further reflected in a shift in responsive *B56* subclasses: *B56-η* members (*FaPP2AB′η-5*, *-9*, *-10*) dominated the 24 h response, while *B56-κ* members (*FaPP2AB′κ-1*, *-2*) became more prominent at later time points. This subclass-specific temporal regulation may provide a mechanism for fine-tuning PP2A substrate specificity at different stages of stress adaptation: the early induction of *B56-η* may initiate the acute antioxidant response, while the later activation of *B56-κ* may sustain long-term stress adaptation or contribute to signal termination. Such temporal partitioning of B subunit function has been proposed in other systems [11]. The 24 h qRT-PCR results corroborated the RNA-seq expression patterns of the top ALA-enhanced genes; extending this validation to the 48 h and 72 h time points will be an important direction for future work.

### 4.5. Functional Divergence of C2 and C4 Catalytic Subunits Under Salt Stress

A notable finding of our transcriptome analysis was the contrasting expression patterns of the two *PP2A* catalytic subclasses, *C2* and *C4*, in response to salt stress. In the descriptive FPKM-based comparison, *C2* members were generally upregulated by NaCl, whereas *C4* members showed a downward trend, with four of eight members showing log_2_FC values between −0.80 and −0.40. This pattern raises the possibility that the two catalytic subclasses have diverged functionally following their duplication in the plant lineage [54,56], although this requires protein-level confirmation.

The C2 subclass is phylogenetically closer to the canonical mammalian PP2A catalytic subunit and is considered the predominant catalytic isoform in most plant tissues. Its upregulation under salt stress is consistent with an increased demand for PP2A phosphatase activity during stress adaptation, presumably to dephosphorylate stress-activated substrates in the antioxidant defense and ion homeostasis pathways. Within C2, only *FaPP2AC2-6* was nominally increased by ALA; the remaining C2 members did not change significantly. Although this observation rests on a single gene, it suggests that ALA may modulate specific C2 paralogs rather than activating all C2 isoforms, possibly through distinct promoter architectures or differential pairing preferences with B subunits. It is possible that the ALA-responsive B56-η subunits and C2 catalytic subunits associate in the same holoenzymes, but the present data cannot distinguish complex composition from a shared transcriptional response. This possibility should be treated as a hypothesis that requires protein interaction data to test.

In stark contrast, the *C4* subclass was markedly suppressed by NaCl stress, in the descriptive FPKM-based comparison, four of eight members (FaPP2AC4-5, -6, -7, -8) showed log_2_FC values between −0.80 and −0.40, while the remaining *C4* members were largely unchanged. *C4* has been proposed to represent a plant-specific catalytic subunit lineage that diverged from *C2* early in plant evolution. Although less characterized than *C2*, *C4* has been implicated in specific developmental processes and may possess distinct substrate preferences or regulatory properties. In *Arabidopsis*, C4 (PP2AC4/PP2A-4) has been proposed to interact with regulatory subunits distinct from those binding C2, supporting the notion that C2 and C4 form functionally distinct PP2A subpopulations. The coordinated downregulation of C4 under salt stress, together with differential ALA response of C2 members, suggests that PP2A activity may be fine-tuned through combinatorial control of catalytic isoform expression during salt stress. However, whether these transcriptional changes reflect functional divergence at the protein level remains to be determined by direct PP2A activity measurements and substrate identification.

### 4.6. ALA-PP2A-Antioxidant Axis in Salt Tolerance

This study provides evidence for a functional link between ALA, PP2A, and the antioxidant defence system. Three lines of evidence support this model: (1) transcriptome data showing that ALA increases the transcript abundance of B56-η regulatory subunits and one C2 catalytic subunit under NaCl stress; (2) physiological data demonstrating elevated PP2A enzyme activity, enhanced antioxidant enzyme activities, and reduced ROS accumulation with ALA treatment; and (3) pharmacological evidence showing the PP2A inhibitor cantharidin [57] largely attenuates ALA’s protective effects. Our findings complement the recent discovery that ALA enhances strawberry salt tolerance through a NO–H_2_O_2_ signaling circuit regulated by *FaWRKY70* and *FaWRKY40* [27]. Our data do not distinguish whether PP2A acts independently of, or together with, the WRKY-mediated pathway; this remains to be tested. Furthermore, the recent demonstration that MdPP2AC directly interacts with the vacuolar chloride channel MdCLC-c2 to mediate ALA-induced salt tolerance in apple [20] provides independent evidence from another *Rosaceae* species that PP2A is involved in ALA signalling. Together, these findings across strawberry and apple suggest a conserved ALA–PP2A signaling module in the *Rosaceae* family, in which PP2A may contribute to antioxidant defence and ion homeostasis under salt stress.

The cantharidin experiments are consistent with a contribution of PP2A activity to the protective effects of ALA. Cantharidin co-treatment with NaCl + ALA returned most physiological parameters to NaCl-stress levels or below, which suggests that PP2A activity contributes to the manifestation of ALA’s protective effects. Cantharidin also inhibits protein phosphatase 1, however, and this experiment therefore cannot distinguish between the two activities; no genetic validation was performed in this study. The result should be regarded as supportive rather than definitive. This finding is consistent with genetic evidence from *Arabidopsis*, where B″ and C subunit mutants exhibit altered ROS levels and SOD activities, indicating that PP2A modulates antioxidant enzyme function at the post-translational level [58].

Our analysis shows that ALA alters the transcript abundance of specific PP2A subunits under salt stress, notably increasing *FaPP2AB′η-5* and *FaPP2AB′η-9* and decreasing *FaPP2AB′κ-1*, rather than uniformly upregulating the family. Because B subunits contribute to substrate specificity, this pattern raises the possibility that the composition of PP2A holoenzymes shifts in favour of B56-η-containing complexes. However, we provide protein-level data showing that total PP2A increases under salt stress and is further increased by ALA (Figure 7E). This supports the transcript-level findings at the protein level. Nevertheless, the immunoassay is not subunit-specific and does not report holoenzyme composition. The preferential association of B56-η with C2 subunits therefore remains a hypothesis, and protein interaction data, for example, co-immunoprecipitation or bimolecular fluorescence complementation, will be required to test it.

Several molecular mechanisms may explain how PP2A activation leads to enhanced antioxidant defense. At the post-translational level, PP2A may directly dephosphorylate and modulate the activity of antioxidant enzymes and ion transporters. *Arabidopsis* phosphoproteomic studies have identified SOD and CAT as phosphorylated proteins [59,60], and PP2AB″ and C subunit mutants exhibit altered SOD activity, providing genetic evidence that PP2A regulates antioxidant enzyme function [58]. In *Arabidopsis* and apple, PP2A catalytic subunits directly interact with vacuolar chloride channels (CLC-c2/CLCa/CLCc) to promote ion sequestration under salt stress [13,20], providing robust evidence for post-translational regulation of ion homeostasis by PP2A. At the transcriptional level, PP2A B subunits interact with stress-responsive transcription factors in ethylene and immune response pathways to modulate gene expression [11]. Additionally, PP2A may participate in feedback regulation of ROS signaling: in the wild diploid strawberry *Fragaria viridis*, the PP2A catalytic subunit FviPP2Ac directly binds and inhibits NADPH oxidase FviRbohH [61], suggesting a conserved PP2A-ROS regulatory module in Fragaria that remains to be validated in cultivated strawberry under salt stress.

The ion data provide a direct physiological readout of the ALA response under salt stress. ALA reduced Na^+^ accumulation, increased K^+^ content in roots, and lowered the Na^+^/K^+^ ratio in both leaves and roots relative to NaCl alone, indicating that ALA helps to maintain ionic homeostasis. This is consistent with earlier reports that ALA improves ion homeostasis under salinity in several crop species [24,25,26]. Importantly, co-application of cantharidin with ALA reversed these effects in both tissues, so the ion data parallel the growth, antioxidant and oxidative-damage parameters. Together with the reported interaction between PP2A catalytic subunits and vacuolar chloride channels, these results support a contribution of PP2A activity to ion homeostasis during the ALA response to salt stress.

### 4.7. Limitations

This study has several limitations. First, the original FASTQ files from the RNA-seq experiment are no longer available, so read-level quality metrics such as sequencing depth, mapping rate and the proportion of uniquely mapped reads cannot be reported. Sample-level reproducibility was therefore assessed from the expression matrix: a principal component analysis of all 21 samples and a pairwise correlation analysis of the 24 h samples (Figure S1) showed high correlation between biological replicates. Second, a count matrix was available only for the NaCl and NaCl + ALA samples at 24 h, so the DESeq2 analysis is restricted to that contrast; the NaCl versus CK comparison and the 48 h and 72 h time points remain descriptive. Third, one NaCl + ALA sample at 24 h was excluded after library preparation failed, so the 24 h NaCl + ALA group comprises two biological replicates; despite this, *FaPP2AB′η-5* reached an FDR-adjusted *p* value of 2.7 × 10^−5^. Fourth, the RNA-seq experiment did not include an ALA-only treatment, so the transcriptional changes reported here describe the effect of ALA under salinity and cannot be attributed to ALA signalling alone. Fifth, the pharmacological evidence rests on cantharidin, which inhibits both PP2A and protein phosphatase 1, and no genetic validation was performed. The nomenclature used here rests on sequence similarity to the closest *Arabidopsis* homologues, and no subgenome-resolved comparison with the diploid progenitor genomes was performed, so the assignment does not establish orthology. Finally, the PP2A immunoassay is not subunit-specific and the antibody pair has not been independently validated in strawberry, so it reports total PP2A rather than the activity of a defined holoenzyme.

## 5. Conclusions

This study provides a comprehensive characterization of the *PP2A* gene family in strawberry, identifying 75 members distributed across five subfamilies. Segmental duplication driven by polyploidization is the primary mechanism of family expansion, with all members under strong purifying selection. Promoter analysis identified a diverse repertoire of stress- and hormone-responsive cis-acting elements in silico. Through integrated transcriptomic and pharmacological analyses, we show that ALA increases the transcript abundance of specific *B56-η* regulatory subunits of PP2A in a time-dependent manner and elevates PP2A activity under NaCl stress, while *C4* catalytic subunits are transcriptionally suppressed. These changes are accompanied by improved ion homeostasis, increased antioxidant enzyme activities and reduced oxidative damage. Whether they reflect a coordinated change in PP2A holoenzyme composition remains to be tested. The PP2A inhibitor cantharidin largely attenuated ALA-mediated protection, consistent with a contribution of PP2A activity to the antioxidant-promoting effects of ALA. Because cantharidin also inhibits protein phosphatase 1, this pharmacological result supports but does not establish a specific role for PP2A. These findings reveal a novel ALA-PP2A-antioxidant regulatory axis and provide a theoretical basis for developing ALA-based strategies to improve salt tolerance in strawberry and other crops.

## Data Availability

The original contributions presented in this study are included in the article/Supplementary Materials. Further inquiries can be directed to the corresponding authors.

## Supplementary Materials

The following supporting information can be downloaded at https://www.mdpi.com/article/10.3390/genes17101213/s1. Table S1: Primers used for qRT-PCR validation. Table S2: Reciprocal BLASTP results used for the orthologous naming of *FaPP2A* genes. Table S3: Genomic information of the *75 FaPP2A* genes, protein physicochemical properties, and predicted subcellular localization. Table S4: Threshold sensitivity of the four-quadrant classification of ALA-responsive FaPP2A genes. Figure S1: Quality assessment of the RNA-seq data. Figure S2: Effects of ALA and cantharidin on root ion contents under NaCl stress after 4 days of treatment.

## References

[1] Munns R., Tester M. (2008). Mechanisms of Salinity Tolerance. Annu. Rev. Plant Biol..

[2] Garriga M., Muñoz C.A., Caligari P.D.S., Retamales J.B. (2015). Effect of Salt Stress on Genotypes of Commercial (*Fragaria* × *Ananassa*) and Chilean Strawberry (*F*. *Chiloensis*). Sci. Hortic..

[3] Li S., Chang L., Sun R., Dong J., Zhong C., Gao Y., Zhang H., Wei L., Wei Y., Zhang Y. (2022). Combined Transcriptomic and Metabolomic Analysis Reveals a Role for Adenosine Triphosphate-Binding Cassette Transporters and Cell Wall Remodeling in Response to Salt Stress in Strawberry. Front. Plant Sci..

[4] Edger P.P., Poorten T.J., VanBuren R., Hardigan M.A., Colle M., McKain M.R., Smith R.D., Teresi S.J., Nelson A.D.L., Wai C.M. (2019). Origin and Evolution of the Octoploid Strawberry Genome. Nat. Genet..

[5] Zhu J.-K. (2016). Abiotic Stress Signaling and Responses in Plants. Cell.

[6] Zhu J.-K. (2002). Salt and Drought Stress Signal Transduction in Plants. Annu. Rev. Plant Biol..

[7] De Zelicourt A., Colcombet J., Hirt H. (2016). The Role of MAPK Modules and ABA during Abiotic Stress Signaling. Trends Plant Sci..

[8] Boudsocq M., Sheen J. (2013). CDPKs in Immune and Stress Signaling. Trends Plant Sci..

[9] Janssens V., Goris J. (2001). Protein Phosphatase 2A: A Highly Regulated Family of Serine/Threonine Phosphatases Implicated in Cell Growth and Signalling. Biochem. J..

[10] Farkas I., Dombrádi V., Miskei M., Szabados L., Koncz C. (2007). *Arabidopsis* PPP Family of Serine/Threonine Phosphatases. Trends Plant Sci..

[11] Máthé C., Freytag C., Kelemen A., M-Hamvas M., Garda T. (2023). “B” Regulatory Subunits of PP2A: Their Roles in Plant Development and Stress Reactions. Int. J. Mol. Sci..

[12] Pérez-Callejón E., Casamayor A., Pujol G., Camps M., Ferrer A., Ariño J. (1998). Molecular Cloning and Characterization of Two Phosphatase 2A Catalytic Subunit Genes from *Arabidopsis thaliana*. Gene.

[13] Hu R., Zhu Y., Shen G., Zhang H. (2017). Overexpression of the *PP2A-C5* Gene Confers Increased Salt Tolerance in *Arabidopsis thaliana*. Plant Signal Behav..

[14] Liu J., Wang M., Liu X., Wang X., Li Z., Luo J., Lin W., Zhao C. (2026). FERONIA Kinase and PP2A Antagonistically Regulate Salt Tolerance in *Arabidopsis*. Curr. Biol..

[15] Bhattacharjee S., Paul A., Jana A., Unnati G.M., R. D., Miao Y., Lu H., Shen G., Mishra N. (2025). Genome-Wide Identification of PP2A Gene Family in *Camellia Sinensis* Reveals the Potential Role of *CsPP2A-TON2/FASS1* in Abiotic Stress. Peer J..

[16] Chao J., Huang Z., Yang S., Deng X., Tian W. (2020). Genome-Wide Identification and Expression Analysis of the Phosphatase 2A Family in Rubber Tree (*Hevea brasiliensis*). PLoS ONE.

[17] Xiong Y., Fan X.-H., Wang Q., Yin Z.-G., Sheng X.-W., Chen J., Zhou Y.-B., Chen M., Ma Y.-Z., Ma J. (2022). Genomic Analysis of Soybean *PP2A-B″* Family and Its Effects on Drought and Salt Tolerance. Front. Plant Sci..

[18] Booker M.A., DeLong A. (2017). Atypical Protein Phosphatase 2A Gene Families Do Not Expand via Paleopolyploidization. Plant Physiol..

[19] Chen Z., Wang L. (2022). ALA Upregulates MdPTPA Expression to Increase the PP2A Activity and Promote Stomatal Opening in Apple Leaves. Plant Sci..

[20] Xiang K., Zhou J., Zhang J., Iqbal M., Wang L. (2026). MdPP2AC Interacting with MdCLC to Mediate ALA-Induced Salt Tolerance in Apple. Plant Physiol. Biochem..

[21] Tanaka R., Tanaka A. (2007). Tetrapyrrole Biosynthesis in Higher Plants. Annu. Rev. Plant Biol..

[22] Akram N.A., Ashraf M., Al-Qurainy F. (2012). Aminolevulinic Acid-Induced Changes in Some Key Physiological Attributes and Activities of Antioxidant Enzymes in Sunflower (*Helianthus annuus* L.) Plants under Saline Regimes. Sci. Hortic..

[23] Wu Y., Jin X., Liao W., Hu L., Dawuda M.M., Zhao X., Tang Z., Gong T., Yu J. (2018). 5-Aminolevulinic Acid (ALA) Alleviated Salinity Stress in Cucumber Seedlings by Enhancing Chlorophyll Synthesis Pathway. Front. Plant Sci..

[24] Kaya C., Ashraf M. (2021). Nitric Oxide Is Required for Aminolevulinic Acid-Induced Salt Tolerance by Lowering Oxidative Stress in Maize (*Zea mays*). J. Plant Growth Regul..

[25] Wang X., Yang S., Li B., Chen C., Li J., Wang Y., Du Q., Li M., Wang H., Li J. (2024). Exogenous 5-Aminolevulinic Acid Enhanced Saline-Alkali Tolerance in Pepper Seedlings by Regulating Photosynthesis, Oxidative Damage, and Glutathione Metabolism. Plant Cell Rep..

[26] Jiang Y., Li M., Qian Y., Rong H., Xie T., Wang S., Zhao H., Yang L., Wang Q., Cao Y. (2025). Analysis of the Transcriptome Provides Insights into the Photosynthate of Maize Response to Salt Stress by 5-Aminolevulinic Acid. Int. J. Mol. Sci..

[27] Yang H., Zhang J., Zhong Y., Wang L. (2025). 5-Aminolevulinic Acid Improves Strawberry Salt Tolerance through a NO–H_2_O_2_ Signaling Circuit Regulated by FaWRKY70 and FaWRKY40. J. Adv. Res..

[28] Jung S., Lee T., Cheng C.-H., Buble K., Zheng P., Yu J., Humann J., Ficklin S.P., Gasic K., Scott K. (2019). 15 Years of GDR: New Data and Functionality in the Genome Database for Rosaceae. Nucleic Acids Res..

[29] Lamesch P., Berardini T.Z., Li D., Swarbreck D., Wilks C., Sasidharan R., Muller R., Dreher K., Alexander D.L., Garcia-Hernandez M. (2012). The *Arabidopsis* Information Resource (TAIR): Improved Gene Annotation and New Tools. Nucleic Acids Res..

[30] Altschul S. (1997). Gapped BLAST and PSI-BLAST: A New Generation of Protein Database Search Programs. Nucleic Acids Res..

[31] Mistry J., Chuguransky S., Williams L., Qureshi M., Salazar G.A., Sonnhammer E.L.L., Tosatto S.C.E., Paladin L., Raj S., Richardson L.J. (2021). Pfam: The Protein Families Database in 2021. Nucleic Acids Res..

[32] Eddy S.R. (2011). Accelerated Profile HMM Searches. PLoS Comput. Biol..

[33] Jones P., Binns D., Chang H.-Y., Fraser M., Li W., McAnulla C., McWilliam H., Maslen J., Mitchell A., Nuka G. (2014). InterProScan 5: Genome-Scale Protein Function Classification. Bioinformatics.

[34] Lu S., Wang J., Chitsaz F., Derbyshire M.K., Geer R.C., Gonzales N.R., Gwadz M., Hurwitz D.I., Marchler G.H., Song J.S. (2020). CDD/SPARCLE: The Conserved Domain Database in 2020. Nucleic Acids Res..

[35] Horton P., Park K.-J., Obayashi T., Fujita N., Harada H., Adams-Collier C.J., Nakai K. (2007). WoLF PSORT: Protein Localization Predictor. Nucleic Acids Res..

[36] Edgar R.C. (2004). MUSCLE: Multiple Sequence Alignment with High Accuracy and High Throughput. Nucleic Acids Res..

[37] Tamura K., Stecher G., Kumar S. (2021). MEGA11: Molecular Evolutionary Genetics Analysis Version 11. Mol. Biol. Evol..

[38] Letunic I., Bork P. (2021). Interactive Tree Of Life (iTOL) v5: An Online Tool for Phylogenetic Tree Display and Annotation. Nucleic Acids Res..

[39] Chen C., Chen H., Zhang Y., Thomas H.R., Frank M.H., He Y., Xia R. (2020). TBtools: An Integrative Toolkit Developed for Interactive Analyses of Big Biological Data. Mol. Plant.

[40] Bailey T.L., Boden M., Buske F.A., Frith M., Grant C.E., Clementi L., Ren J., Li W.W., Noble W.S. (2009). MEME SUITE: Tools for Motif Discovery and Searching. Nucleic Acids Res..

[41] Wang Y., Tang H., De Barry J.D., Tan X., Li J., Wang X., Lee T.-H., Jin H., Marler B., Guo H. (2012). MCScanX: A Toolkit for Detection and Evolutionary Analysis of Gene Synteny and Collinearity. Nucleic Acids Res..

[42] Lescot M. (2002). PlantCARE, a Database of Plant Cis-Acting Regulatory Elements and a Portal to Tools for in Silico Analysis of Promoter Sequences. Nucleic Acids Res..

[43] Katoh K., Standley D.M. (2013). MAFFT Multiple Sequence Alignment Software Version 7: Improvements in Performance and Usability. Mol. Biol. Evol..

[44] Nei M., Gojobori T. (1986). Simple Methods for Estimating the Numbers of Synonymous and Nonsynonymous Nucleotide Substitutions. Mol. Biol. Evol..

[45] Wang D., Zhang Y., Zhang Z., Zhu J., Yu J. (2010). KaKs_Calculator 2.0: A Toolkit Incorporating Gamma-Series Methods and Sliding Window Strategies. Genom. Proteom. Bioinf..

[46] Kim D., Paggi J.M., Park C., Bennett C., Salzberg S.L. (2019). Graph-Based Genome Alignment and Genotyping with HISAT2 and HISAT-Genotype. Nat. Biotechnol..

[47] Pertea M., Pertea G.M., Antonescu C.M., Chang T.-C., Mendell J.T., Salzberg S.L. (2015). StringTie Enables Improved Reconstruction of a Transcriptome from RNA-Seq Reads. Nat. Biotechnol..

[48] Livak K.J., Schmittgen T.D. (2001). Analysis of Relative Gene Expression Data Using Real-Time Quantitative PCR and the 2^−ΔΔCT^ Method. Methods.

[49] Yang H., Wu F., Cheng J. (2011). Reduced Chilling Injury in Cucumber by Nitric Oxide and the Antioxidant Response. Food Chem..

[50] Cui G., Zhao X., Liu S., Sun F., Zhang C., Xi Y. (2017). Beneficial Effects of Melatonin in Overcoming Drought Stress in Wheat Seedlings. Plant Physiol. Biochem..

[51] Bates L.S., Waldren R.P., Teare I.D. (1973). Rapid Determination of Free Proline for Water-Stress Studies. Plant Soil.

[52] DuBois M., Gilles K.A., Hamilton J.K., Rebers P.A., Smith F. (1956). Colorimetric Method for Determination of Sugars and Related Substances. Anal. Chem..

[53] Singh A., Giri J., Kapoor S., Tyagi A.K., Pandey G.K. (2010). Protein Phosphatase Complement in Rice: Genome-Wide Identification and Transcriptional Analysis under Abiotic Stress Conditions and Reproductive Development. BMC Genom..

[54] Lynch M., Conery J.S. (2000). The Evolutionary Fate and Consequences of Duplicate Genes. Science.

[55] Spinner L., Gadeyne A., Belcram K., Goussot M., Moison M., Duroc Y., Eeckhout D., De Winne N., Schaefer E., Van De Slijke E. (2013). A Protein Phosphatase 2A Complex Spatially Controls Plant Cell Division. Nat. Commun..

[56] Force A., Lynch M., Pickett F.B., Amores A., Yan Y., Postlethwait J. (1999). Preservation of Duplicate Genes by Complementary, Degenerative Mutations. Genetics.

[57] Honkanen R.E. (1993). Cantharidin, Another Natural Toxin That Inhibits the Activity of Serine/Threonine Protein Phosphatases Types 1 and 2A. FEBS Lett..

[58] Freytag C., Garda T., Kónya Z., M-Hamvas M., Tóth-Várady B., Juhász G.P., Ujlaky-Nagy L., Kelemen A., Vasas G., Máthé C. (2023). B″ and C Subunits of PP2A Regulate the Levels of Reactive Oxygen Species and Superoxide Dismutase Activities in *Arabidopsis*. Plant Physiol. Biochem..

[59] Roitinger E., Hofer M., Köcher T., Pichler P., Novatchkova M., Yang J., Schlögelhofer P., Mechtler K. (2015). Quantitative Phosphoproteomics of the Ataxia Telangiectasia-Mutated (ATM) and Ataxia Telangiectasia-Mutated and Rad3-Related (ATR) Dependent DNA Damage Response in *Arabidopsis thaliana*. Mol. Cell. Proteom..

[60] Mergner J., Frejno M., List M., Papacek M., Chen X., Chaudhary A., Samaras P., Richter S., Shikata H., Messerer M. (2020). Mass-Spectrometry-Based Draft of the Arabidopsis Proteome. Nature.

[61] Ni Z., Pan J., Zou Y., Pan Z., Wang T., Xu L., Shen Z., Wu E., Qiao Y. (2025). S-RNase-Mediated Attenuation of FviPP2Ac-FviRbohH Binding Promotes ROS Accumulation and Arrests Pollen Tube Growth in Fragaria Viridis. Plant Physiol. Biochem..

