# Supplementary material for: Genome-Wide Identification of the PP2A Gene Family in Strawberry (Fragaria × ananassa) and Pharmacological Evidence for the Involvement of PP2A in ALA-Mediated Salt Tolerance
Source: Genes (Basel). 2026 Sep 30;17(10):1213. doi: 10.3390/genes17101213 (PMC13704014; doi:10.3390/genes17101213)
Supplement: Supplementary file 1 [file genes-17-01213-s001.zip › genes-4589099-supplementary/Figures S1 and S2.pdf]

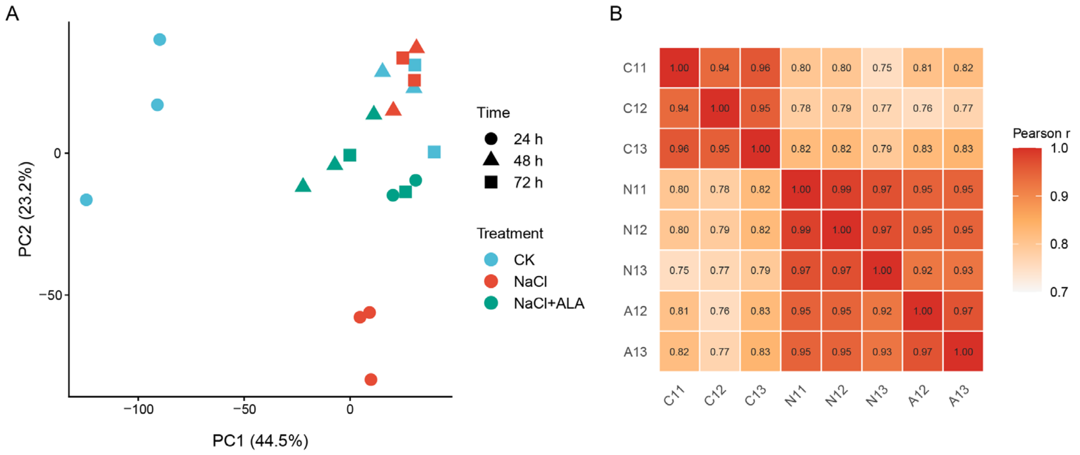

Figure S1. Quality assessment of the RNA-seq data.

(A) Principal component analysis of all 21 RNA-seq samples, based on the 3,000 most variable genes after $\log_2$(FPKM+1) transformation. Genes with FPKM > 1 in fewer than three samples were excluded. PC1 and PC2 account for 44.5% and 23.2% of the variance, respectively. Samples are coloured by treatment and shaped by time point. (B) Pairwise Pearson correlation of log2(FPKM+1) values among the eight samples of the 24 h time point. Numbers in the cells are correlation coefficients. Biological replicates within the same treatment were highly correlated (CK, 0.94-0.96; NaCl, 0.97-0.99; NaCl+ALA, 0.97), whereas samples from different treatments were less correlated (CK versus NaCl or NaCl+ALA, 0.75-0.84).

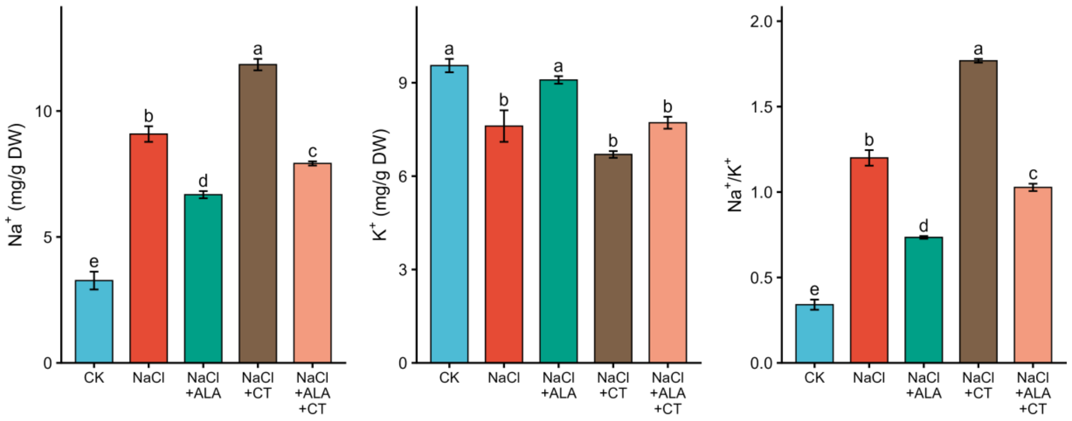

Figure S2. Effects of ALA and cantharidin on root ion contents under NaCl stress after 4 days of treatment.

(A) Root Na^+ content. (B) Root K^+ content. (C) Root Na^+/K^+ ratio. Data are presented as mean ± SD (n = 3). Different lowercase letters indicate significant differences at $P < 0.05$ by one-way ANOVA followed by Tukey's HSD test. DW, dry weight.
